# Reduced expression of transforming growth factor-beta receptor type III in high stage neuroblastomas

**DOI:** 10.1054/bjoc.1999.1058

**Published:** 2000-02-21

**Authors:** A Iolascon, L Giordani, A Borriello, R Carbone, A Izzo, G P Tonini, C Gambini, F Della Ragione

**Affiliations:** Department of ‘Biomedicina dell’Età Evolutiva', University of Bari, Piazza G. Cesàce 11, Bari, 70124, Italy; Institute of Biochemistry of Macromolecules, Second University of Naples, Italy; Divison of Pathology, Giannina Gaslini Children's Hospital, Genova, Italy; Advanced Biotechnology Center, Genova, Italy

**Keywords:** TGF-β1, TGF-βRI, TGF-βRII, TGF-βRIII, neuroblastoma

## Abstract

Transforming growth factor beta (TGF-β) is a powerful inhibitor of cell proliferation and a potent inducer of differentiation. Resistance to TGF-β action is a characteristic of many malignancies and has been attributed to alterations of TGF-β receptors as well as disturbance of downstream transduction pathways. To analyse the TGF-β response in neuroblastoma, the expression of TGF-β1 and TGF-β type I, II and III receptor genes was investigated in 61 cancer samples by means of reverse transcription polymerase chain reaction. The specimens analysed belong to different stages, namely nine samples of stage 1, ten of stage 2, nine of stage 3 and 28 of stage 4. Moreover, five samples were of stage 4S, which represents a tumour form undergoing spontaneous regression. The results obtained show that TGF-β1 and TGF-β type I and II receptor genes appear to be almost equally expressed in neuroblastomas of all stages. Conversely, TGF-β type III receptor gene expression, which is required for an efficacious TGF-β binding and function, is strongly reduced exclusively in neuroblastomas of stages 3 and 4. These findings were directly confirmed by immunohistochemical analyses of ten neuroblastoma specimens. Our results suggest the occurrence of an altered TGF-β response in advanced neuroblastomas which might be an important mechanism for escaping growth control and for developing invasiveness. Moreover, our findings allow the proposal of a novel mechanism, namely down-regulation of TGF-β type III receptor gene expression, to avoid TGF-β inhibitory activity. © 2000 Cancer Research Campaign


British Journal of Cancer (2000) 82(6), 1171–1176
© 2000 Cancer Research Campaign
Article no. bjoc.1999.1058, available online at http://www.idealibrary.com on IDEAL®

### Reduced expression of transforming growth factor-beta receptor type III in high stage neuroblastomas

**A Iolascon[1], L Giordani[1], A Borriello[2], R Carbone[1], A Izzo[1], GP Tonini[4], C Gambini[3] and F Della Ragione[2]**

[1]Department of 'Biomedicina dell'Età Evolutiva', University of Bari, Piazza G. Cesaȅe 11, 70124 Bari, Italy; [2]Institute of Biochemistry of Macromolecules, Second University of Naples, Italy; [3]Divison of Pathology, Giannina Gaslini Children's Hospital, Genova, Italy; [4]Advanced Biotechnology Center, Genova, Italy

**Summary** Transforming growth factor beta (TGF-β) is a powerful inhibitor of cell proliferation and a potent inducer of differentiation. Resistance to TGF-β action is a characteristic of many malignancies and has been attributed to alterations of TGF-β receptors as well as disturbance of downstream transduction pathways. To analyse the TGF-β response in neuroblastoma, the expression of TGF-β1 and TGF-β type I, II and III receptor genes was investigated in 61 cancer samples by means of reverse transcription polymerase chain reaction. The specimens analysed belong to different stages, namely nine samples of stage 1, ten of stage 2, nine of stage 3 and 28 of stage 4. Moreover, five samples were of stage 4S, which represents a tumour form undergoing spontaneous regression. The results obtained show that TGF-β1 and TGF-β type I and II receptor genes appear to be almost equally expressed in neuroblastomas of all stages. Conversely, TGF-β type III receptor gene expression, which is required for an efficacious TGF-β binding and function, is strongly reduced exclusively in neuroblastomas of stages 3 and 4. These findings were directly confirmed by immunohistochemical analyses of ten neuroblastoma specimens. Our results suggest the occurrence of an altered TGF-β response in advanced neuroblastomas which might be an important mechanism for escaping growth control and for developing invasiveness. Moreover, our findings allow the proposal of a novel mechanism, namely down-regulation of TGF-β type III receptor gene expression, to avoid TGF-β inhibitory activity. © 2000 Cancer Research Campaign

**Keywords:** TGF-β1; TGF-βRI; TGF-βRII; TGF-βRIII; neuroblastoma

Neuroblastomas derive from the arrested differentiation of neural crest sympathoadrenal progenitor cells. Clinical evidences indicate that this differentiation block is reversible, since a large percentage of localized early-stage cancers and the majority of the congenital disseminated forms of neuroblastoma might undergo spontaneous regression (Siegel and Sato, 1986). On the other hand, high-stage metastatic neuroblastomas (stages 3 and 4) are generally less differentiated and more primitive than their localized stages. However, high-stage neuroblastomas may differentiate, as shown by the ability of their derived cell lines to acquire neuronal cell morphology and markers. Unfortunately, patients with advanced stage neuroblastoma have poor outcome and scarce long-term survival, even after aggressive therapy (Pinkerton, 1993).

On the basis of these findings, it appears important to investigate, in neuroblastoma, the molecular mechanisms involved in the interplay between proliferation and differentiation. In particular, studies on genes which have been demonstrated to play a key function in differentiation are of pivotal relevance.

A pivotal pathway, involved in the control of proliferation/differentiation, requires the activation of transforming growth factor beta (TGF-β) receptors and the cascade of events which finally results in growth impairment and differentiation. TGF-β exerts its effect by interacting mainly with three membrane proteins named type I (RI), type II (RII) and type III (RIII) receptors. TGF-βRI and TGF-βRII form a heterodimeric or heterotetrameric complex (with one to one stoichiometry) necessary for TGF-β signal transduction (Heldin et al, 1997), while TGF-βRIII (also called β-glycan) is required to allow (by increasing the affinity) the binding of TGF-βRII to the various TGF-β isoforms.

A number of studies have investigated the occurrence in human cancers of structural and functional alterations of TGF-βRI and TGF-βRII genes. Mutations of TGF-βRII occur in human malignancies, including retinoblastoma and colon cancers and in cell lines derived from carcinomas of the uterine cervix (Coffey et al, 1987; Kimchi et al, 1988; Lu et al, 1995; Markowitz et al, 1995). Moreover, several investigations demonstrated a lower expression of TGF-βRI and TGF-βRII genes in human malignancies (Kadin et al, 1994; Matoba et al, 1998; Royuela et al, 1998; Hougaard et al, 1999). These results have been confirmed by the observation that the forced expression of TGF-βRI and TGF-βRII genes remarkably reduces malignancy in the recipient cancer cell lines (Sun et al, 1994; Wang et al, 1996).

On the other hand, to the best of our knowledge, only one study on TGF-βRIII transcript in human cancers has been carried out. This very recent investigation demonstrated an impairment of TGF-βRIII expression in ovarian carcinomas (Bristow et al, 1999). In this scenario it is important to note that recently it has also been shown that TGF-βRIII overexpression restores an apparent unresponsiveness of breast cancer cells to the inhibitory action of TGF-β1 (Chen et al, 1997). Thus, down-regulation of TGF-βRIII might be an additional important mechanism of malignant cells for escaping TGF-β-induced growth inhibition and induction of differentiation.

Although TGF-β1 and TGF-β receptor genes are involved in the control of cell differentiation and neuroblastoma development is

*Received 10 May 1999*
*Revised 18 October 1999*
*Accepted 2 November 1999*

*Correspondence to:* A Iolascon linked to differentiation arrest, no studies have been carried out so far to analyse the expression of these four genes in human neuroblastomas. This investigation is peculiarly important in view of the observation that neuronal tissue is responsive to TGF-β control. Moreover, very few investigations have been performed on TGF-βRIII expression in human tumours even though, as discussed above, this gene might also be considered as a potential cancer suppressor gene.

In this report, we described the first investigation on TGF-β and TGF-β receptor (type I, II and III) gene expression in a large series (61 specimens) of human neuroblastoma. This study was carried out in order to evaluate the role of these genes in the development and evolution of such a frequent paediatric tumour. The results obtained were also compared with two other neuroblastoma important genetic features, namely N-*myc* gene amplification and 1p chromosome status.

### MATERIALS AND METHODS

### Tumour samples

Sixty-one primary tumour samples, belonging to the Italian Tissue Bank, were selected to represent the distribution of stages found in neuroblastoma. Patients were staged according to the revised INSS staging system (Brodeur et al, 1993). The resected tumours were frozen immediately in liquid nitrogen and stored at –80°C until analysis. Diagnosis was established by histological examination of tumour tissue obtained at surgery. In order to ensure that tumour samples used for molecular analyses contained a sufficient proportion of malignant cells, several cytological and histological evaluations of each sample were performed. Thus, only samples which were clearly demonstrated to contain more than 95% tumour cells were used in the present study. This allowed us to rule out the possibility that the obtained results were due to normal cells present in the analysed specimens.

### RT-PCR

Reverse transcription polymerase chain reaction (RT-PCR) analysis was performed using the StrataScript RT-PCR Kit (Stratagene, La Jolla, CA, USA). Briefly, 2.5 μg of total RNA, prepared as reported (Iolascon et al, 1998*b*), were reverse-transcribed by StrataScript RNAase H- reverse transcriptase (25 U) using oligo(dT) primer (150 ng) in a final volume of 25 μl. cDNA samples were diluted tenfold in a PCR reaction assay to a volume of 50 μl containing, in addition to the DNA template, 30 mM Tris–HCl (pH 9.0), 50 mM potassium chloride, 1.5 mM magnesium chloride, 200 μg of each primer, 0.2 mM of each nucleotide and 1 unit of *Taq* DNA polymerase. Temperature conditions and primers for glyceraldehyde 3-phosphate dehydrogenase (GAPDH) were previously reported (Iolascon et al, 1998*b*).

Primers used for TGF-β1 were as follows: 5′-TGTCCCCTATCCCCTGACTCCC-3′, and 5′-CCCAGCCTGGAAGGCCTCCATC-3′ (amplified fragment of 221 bp). Temperature conditions for TGF-β1 were: hot start at 95°C for 5 min, 30 cycles composed of steps at 95°C for 1 min, 65°C for 1 min, 72°C for 1 min and a final elongation step at 72°C for 7 min.

Primers used for TGF-βRI were as follows: 5′-ATTCCTCGAGATAGGCCGTT-3′, and 5′-AGGGCGATCTAATGAAGGGT-3′ (amplified fragment of 288 bp). Temperature conditions for TGF-βRI were: hot start at 95°C for 5 min, 30 cycles composed of steps at 95°C for 1 min, 58°C for 1 min, 72°C for 1 min and a final elongation step at 72°C for 7 min.

Primers used for TGF-βRII were as follows: 5′-TGTGTTCCTGTAGCTCTGATG-3′, and 5′-AGATCTTGACTGCCACTGTCTC-3′ (amplified fragment of 432 bp). Temperature conditions for TGF-βRII were: hot start at 94°C for 3 min, 30 cycles composed of steps at 94°C for 1 min, 60°C for 1 min, 72°C for 1 min and a final elongation step at 72°C for 7 min. Primers used for TGF-βRIII were as follows: 5′-TGTCACCTGGCACATTCATT-3′, and 5′-TCTCAGCACTGTCTTGGTGG-3′ (amplified fragment of 246 bp). Temperature conditions for TGF-βRIII were: hot start at 94°C for 3 min, 30 cycles composed of steps at 94°C for 1 min, 57°C for 1 min, 72°C for 1 min and a final elongation step at 72°C for 7 min.

Before amplification with each specific primer pairs, an aliquot of the cDNA preparation was amplified using GAPDH primers to determine the efficacy of the generated cDNA. Moreover, we used five different cDNA concentrations to assure that signals (both of GAPDH and of the analysed genes) were proportional to input mRNA. These controls are important for comparison between samples because they ensure that equivalent amounts of RNA are amplified. Finally, each experiment was performed at least in duplicate and, in several cases, in triplicate.

Aliquots of PCR reactions were separated and analysed by electrophoresis on 2% (w/v) agarose gels or non-denaturing 8% (w/v) polyacrylamide gels (acrylamide/bisacrylamide, 29/1). In the latter case, the amplified products were stained using silver nitrate method. In several cases, the amplified products were recovered from the gels and sequenced as reported in Iolascon et al (1998*b*). In all cases the sequence of the amplified products corresponded to that reported in the literature.

### Immunohistochemistry

In order to evaluate the TGF-βRIII protein level, an immunohistochemical study was carried out on the ten available specimes of neuroblastoma cases already analysed by RT-PCR. The analyses were performed on histological slides from formalin-fixed and paraffin-embedded tumour tissues by using goat polyclonal antibodies raised against the carboxyl terminus of human TGF-βRIII (Santa Cruz, sc-6189). Deparaffinized sections underwent a microwave exposition, in 10 mM citrate buffer, pH 6, to retrieve antigenicity and thereafter were treated with 3% hydrogen peroxide for endogenous peroxidase inactivation. After incubation for 30 min at room temperature with 1:1000 dilution of primary antibody, the reaction was underscored using a 1:500 diluted rabbit anti-goat antibody at room temperature for 30 min. The immunocomplexes were visualized by using the Envision system (Dako) following manufacturer's instructions. Colourimetric reaction was accomplished using diamminobenzidine as a chromogen.

### RESULTS

Table 1 reports the stage classification and some genetic features of the neuroblastomas analysed. A total of 61 different specimens were studied, classified into five stages (1–4 and 4S) on the basis of well established clinical criteria. In particular, our investigation involved nine tumours of stage 1, ten of stage 2, nine of stage 3, 28 of stage 4 and five of stage 4S. A large percentage of the specimens

*© 2000 Cancer Research Campaign*

**Table 1** Expression of TGF-β1 and TGF-β receptor type I, II and III genes in human neuroblastoma of different stages

| Sample no. | TGF-βRI | TGF-βRII | TGF-βRIII | TGF-β1 | GAPDH | N-*myc* | 1p status |
|---|---|---|---|---|---|---|---|
| Stage 4S | | | | | | | |
| 586 | + | + | ++ | + | + | 1 | Undel. |
| 809 | +++ | +++ | ++ | + | + | 1 | Tripl./undel. |
| 912 | +++ | ++ | ++ | – | + | 1 | Undel. |
| 803 | +++ | +++ | ++ | ++ | + | 1 | Tripl./undel. |
| 887 | +++ | +++ | + | + | + | 1 | – |
| Stage 1 | | | | | | | |
| 655 | – | + | +++ | ND | + | 1 | Undel. |
| 591 | +++ | ++ | ++ | + | + | 1 | Undel. |
| 823 | +++ | +++ | + | + | + | 1 | Undel. |
| 776 | +++ | – | ++ | – | + | 1 | Undel. |
| 772 | – | + | + | + | + | 1 | Dipl./del. |
| 806 | +++ | – | ++ | – | + | 1 | Tripl./del. |
| 859 | – | – | ++ | + | + | 1 | Tripl./undel. |
| 916 | + | + | ++ | ++ | + | 1 | – |
| 778 | +++ | ++ | – | + | + | 1 | Del. |
| Stage 2 | | | | | | | |
| 606 | +++ | + | + | + | + | 1 | Undel. |
| 652 | +++ | ++ | + | + | + | 1 | Undel. |
| 647 | +++ | ++ | + | + | + | 1 | Undel. |
| 634 | +++ | +++ | + | + | + | 1 | Undel. |
| 852 | +++ | + | + | – | + | 1 | Undel. |
| 851 | +++ | ++ | ++ | – | + | 1 | Undel. |
| 762 | + | – | – | – | + | 1 | Tripl./undel. |
| 764 | +++ | + | – | – | + | 1 | Undel. |
| 656 | + | ++ | – | – | + | 1 | Undel. |
| 626 | +++ | +++ | ++ | + | + | 1 | Undel. |
| Stage 3 | | | | | | | |
| 619 | +++ | + | – | + | + | 13 | Del. 1p32–36 |
| 579 | +++ | + | – | – | + | 1 | Undel. |
| 592 | +++ | – | – | – | + | 12 | Del. 1p32–36 |
| 572 | + | ++ | – | + | + | 1 | Del 1p32 |
| 858 | + | + | – | + | + | 1 | Tripl./undel. |
| 802 | +++ | + | – | | + | 60 | Dipl./undel. |
| 799 | +++ | + | ++ | +++ | 1 | Undel. | |
| 793 | +++ | ++ | – | + | + | 1 | Undel. |
| 698 | + | +++ | ++ | + | + | 1 | – |
| Stage 4 | | | | | | | |
| 687 | – | – | – | + | + | 20 | Undel. |
| 640 | +++ | ++ | – | + | + | 1 | Undel. |
| 691 | + | +++ | – | ++ | + | 1 | Undel. |
| 651 | – | – | – | + | + | 7 | Del. 1p32–36 |
| 945 | ND | +++ | ++ | ND | + | Ampl. | Undel. |
| 718 | +++ | +++ | + | + | + | 1 | – |
| 811 | +++ | +++ | + | + | + | 1 | Undel. |
| 870 | + | – | – | + | + | 1 | – |
| 871 | +++ | +++ | – | + | + | 1 | Undel. |
| 872 | +++ | +++ | + | +++ | + | 1 | Tetr./undel. |
| 876 | +++ | +++ | + | + | + | 1 | Undel. |
| 882 | ++ | +++ | ++ | +++ | + | 1 | Tripl./undel. |
| 701 | +++ | +++ | ++ | + | + | 1 | – |
| 846 | +++ | ++ | – | – | + | 1 | Undel. |
| 728 | ND | + | + | ND | + | 1 | Undel. |
| 780 | + | ++ | – | + | + | 1 | Undel. |
| 837 | +++ | ++ | – | + | + | 1 | Undel. |
| 834 | +++ | ++ | – | + | + | 1 | Del. |
| 321 | +++ | +++ | – | + | + | Ampl. | Del. |
| 292 | ++ | ++ | – | +++ | + | Ampl. | Del. |
| 505 | +++ | +++ | – | + | + | Ampl. | Del. |
| 350 | +++ | ++ | – | + | + | Ampl. | – |
| 690 | +++ | +++ | – | + | + | Ampl. | Del. |
| 664 | +++ | ++ | – | + | + | Ampl. | Del. |
| 597 | + | ++ | – | + | + | Ampl. | – |
| 753 | +++ | ++ | – | + | + | Ampl. | – |
| 651 | +++ | ++ | – | +++ | + | Ampl. | – |
| 644 | +++ | ++ | – | + | + | Ampl. | – |

N-*myc* reports the number of copies of the gene or if the gene is amplified. The symbols –, +, ++ and +++ represent the relative amount of the PCR product estimated by laser scanner analysis. These results were generally a mean of three different experiments. Del., deleted; Ampl., amplified; Tetr., ternary; Tripl., triplicate; Undel., undeleted.

*© 2000 Cancer Research Campaign*

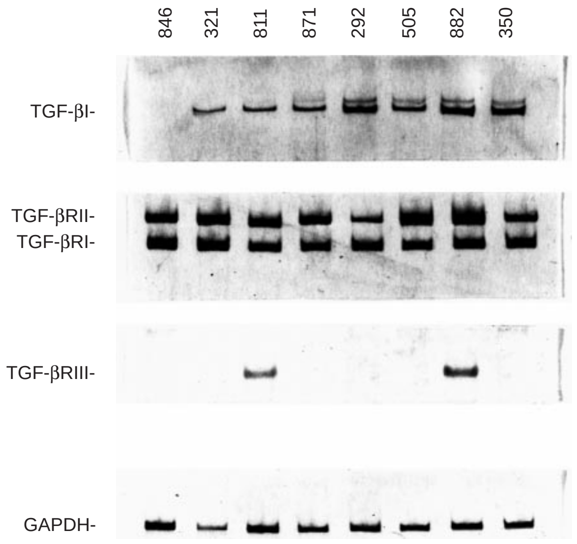

**Figure 1** Analysis of TGF-β1, TGF-βRI, TGF-βRII and TGF-βRII mRNA in human neuroblastoma specimens. Total RNA (2.5 μg) was reverse transcribed as described under Materials and Methods. cDNA samples were then diluted in a PCR assay and amplified by specific primers. Aliquots of PCR reaction (5 μl) were separated by non-denaturing 8% polyacrylamide gel and stained by silver nitrate method. Finally, the relative amount of each amplified product was determined by densitometric scanner. From the top to the bottom: TGF-β1 gene, TGF-βRII gene TGF-βRI, TGF-βRIII gene and GAPDH gene. Numbers reported at the top of panels represent samples examined

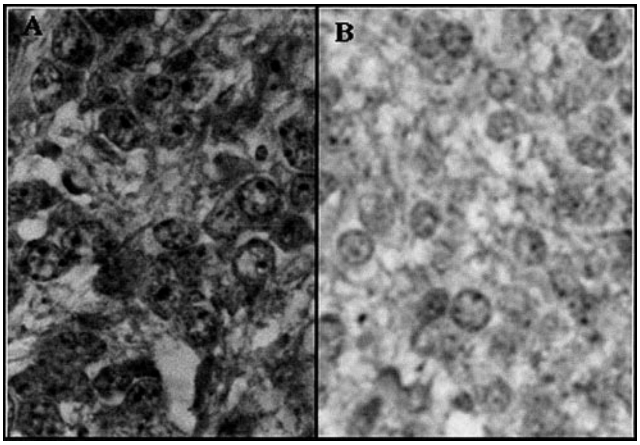

**Figure 2** Immunochemical analysis of TGF-βRIII protein in human neuroblastoma samples. Specimens of neuroblastomas showing high level of TGF-βRIII protein (**A**, sample no. 912) or the absence of the receptor (**B**, sample no. 651). Details on the immunochemical staining technique are reported in Materials and Methods were previously investigated for N-*myc* gene amplification and genetic alteration at 1p level (Iolascon et al, 1998*c*). Each cancer sample was analysed for the expression of TGF-β1, TGF-βRI, TGF-βRII and TGF-βRIII genes by means of RT-PCR methodology. Ten specimens were also studied by immunohistochemistry for TGF-βRIII protein.

**Table 2** mRNA and protein TGF-βRIII level in human neuroblastoma of different stages

| Sample no. | TGF-βRIII mRNA[a] | TGF-βRIII protein (% of positive cells)[b] |
|---|---|---|
| Stage 4S | | |
| 809 | ++ | >75 |
| 912 | ++ | >75 |
| Stage 1 | | |
| 806 | ++ | >75 |
| 859 | ++ | >75 |
| 778 | – | <20 |
| Stage 2 | – | Absent |
| 764 | | |
| Stage 3 | – | Absent |
| 802 | | |
| Stage 4 | | |
| 651 | – | Absent |
| 690 | – | <10 |
| 882 | ++ | >70 |

[a]Determined by RT-PCR. [b]Determined by immunochemical methodologies.

### Expression of TGF-β1 gene and of TGF-βRI, -RII and -RIII genes

Figure 1 shows examples of TGF-β1, TGF-βRI, TGF-βRII and TGF-βRIII gene expression analysed by means of RT-PCR in some neuroblastoma specimens.

TGF-β1 mRNA occurred in neuroblastomas of all stages with small variations (from 60% to 90% of samples from different stages) (Table 1). The gene encoding TGF-βRI and TGF-βRII was expressed in all neuroblastomas of stage 4S and in the majority of specimens belonging to the other stages, including particularly those of stage 4 (Table 1).

The analysis of the transcription of TGF-βRIII gene in stage 4S cancers showed that all specimens (100%) presented the expression of the gene (Table 1). When we investigated TGF-βRIII mRNA in the other neuroblastoma samples we observed an enormous decrease from stage 1 (89%) and stage 2 (70%) to stage 3 (23%) and stage 4 (29%). Importantly, almost all (12 out of 13, 92%) stage 4 and all (three out of three, 100%) stage 3 cancers with N-*myc*-amplified gene did not express TGF-βRIII gene.

### Immunohistochemistochemical analyses

Table 2 reports the results of the immunohistochemical investigation carried out on ten specimens of neuroblastoma. We analysed two samples of neuroblastomas of stage 4S, three of stage 1, one of stages 2 and 3 respectively, and three specimens of stage 4. In the last case, we studied one sample containing high levels of TGF-βRIII gene mRNA and two specimens that do not contain this mRNA. The data obtained, which were reported as percentage of positive cells, strongly confirm, at protein level, the results of RT-PCR studies. Figure 2 shows examples of two neuroblastoma samples analysed by specific TGF-βRIII antibodies. It is evident from the images the occurrence of strong signals, in the positive cells, at cellular membrane level.

 *© 2000 Cancer Research Campaign*

### DISCUSSION

Cell growth and differentiation are two fundamental aspects of multicellular existence and, intertwined with these processes, is the phenomenon of unlimited growth, which is the basis of the neoplastic state. Indeed, cancer might be envisaged as the result of unregulated proliferation of a given cell, frequently due to a block of its ability to undergo differentiation. The present paper reports the results of a study aimed to investigate the expression, in human neuroblastomas, of genes involved in the regulation of TGF-β pathway which plays a key role in the control of the molecular interplay between proliferation and differentiation. All the analysed samples contained more than 95% malignant cells, thus our findings were not (or scarcely) influenced by the occurrence of normal cells in the specimens.

Although TGF-β is one of the most potent inhibitors of cell growth, several malignancies of different origin are resistant to TGF-β, suggesting that the developing of unresponsiveness to this molecule plays an important role in cancerogenesis (Polyak, 1966). Tumours acquire resistance to TGF-β relatively late during malignant progression and this appears to be associated with developing invasiveness (Filmus and Kerbel, 1993; Fynan and Reiss, 1993). Although the loss of TGF-βRII has been reported in retinoblastomas and certain colon carcinoma cell lines (Coffey et al, 1987; Kimchi et al, 1988), it occurs quite infrequently. Conversely, decreased expression of TGF-βRII has been frequently observed in human tumours and cell lines (Filmus et al, 1992; Kadin et al, 1994; Matoba et al, 1998; Royuela et al, 1998; Hougaard at al, 1999), and this phenomenon may result in resistance to TGF-β growth inhibitory activity. When we analysed the TGF-βRII gene expression in human neuroblastomas, we observed that all the neoplasias of stage 4S and the large majority of neuroblastomas of the other stages expressed this specific transcript (Table 1). Thus, these results argue against down-regulation of TGF-βRII expression as an important factor in neuroblastoma development and/or progression. Moreover, no remarkable variation in the expression of TGF-βRI (and of TGF-β1) gene was observable in tumours of different stages.

Very recently it has been demonstrated that TGF-βRIII is also critical in the response to various TGF-β isoforms, and that its overexpression might restore the growth inhibitory effect of TGF-β in the presence of low level TGF-βRII (Chen et al, 1997). Thus, the membrane content of TGF-βRIII is important in TGF-β growth control.

Our results show that TGF-βRIII gene expression is significantly reduced in neuroblastomas of stage 3 and 4. Conversely, all cancers of stage 4S (which spontaneoulsy undergo differentiation) and most stage 1 and 2 tumours express remarkable levels of the TGF-βRIII mRNA. These findings were obtained by employing a quantitative RT-PCR technique and confirmed (on the available samples) by direct immunohistochemical analysis. Thus, the data reported in the present paper demonstrate, for the first time, that neuroblastomas with a worst prognosis and high invasiveness show down-regulation of TGF-βRIII gene transcription. Although the molecular mechanism responsible for this phenomenon is not known and is currently under investigation, it is totally conceivable that the absence of TGF-βRIII might result in an escape of TGF-β antiproliferative action.

TGF-βRIII (also known as β-glycan) gene belongs to a growth factor receptor category which is not coupled to cytoplasmatic structures involved in signalling pathways. This family also includes the p75 gene (encoding a low affinity receptor for nerve growth factor and neurotrophins) (Johnson et al, 1986; Radeke et al, 1987), syndecan and other proteoglycans (whose heparin sulphate chains bind fibroblast growth factors) (Saunders et al, 1989; Kiefer et al, 1990) and the type II receptor for insulin-like growth factors (Morgan et al, 1987; MacDonald et al, 1988). These molecules are of lower affinity and are generally more abundant than the corresponding signalling receptors, properties that might allow them to act as enhancers of growth factor access to the receptors (Lòpez-Casillas et al, 1993). β-glycan is a membrane proteoglycan with heparin and chondrotin sulphate chains attached to a 100 kDa core protein (Lòpez-Casillas et al, 1991; Wang et al, 1991). This protein forms a ternary complex with a TGF-β molecule and the TGF-βRII (Lòpez-Casillas et al, 1993) and increases receptor binding affinity and cell responsiveness to TGF-β (Lòpez-Casillas et al, 1993). For example, in the absence of β-glycan, TGF-βRII binds TGF-β1 with a low affinity ($K_D$ of 0.5 nM) and no detectable affinity for TGF-β2 (Lòpez-Casillas et al, 1993). Conversely, the complex TGF-βRII–TGF-βRIII has an affinity for TGF-β (both type 1 and type 2) in the physiological range (50 pM and lower). Subsequently, the TGF-βRII–TGF-β dimer leaves TGF-βRIII and associates with TGF-βRI forming a ternary complex which activates the signalling pathway. This mechanism establishes that β-glycan membrane level is a direct modulator of TGF-β access to the signalling receptor.

It is well established that retinoic acid induces neuroblastoma cell line differentiation. Moreover, clinical trials are under development in order to evaluate the therapeutical utility in neuroblastoma treatment of retinoic acid and its analogues. It has previously been demonstrated that retinoic-dependent neuroblastoma cell line differentiation is strictly associated with TGF-β secretion and up-regulation of all the TGF-β receptor forms (Cohen et al, 1995). This finding supports the view that the presence of an efficacious TGF-β response is important in neuroblastoma differentiation and that its loss might play a role in the establishment of high-stage neoplasias. Accordingly it is interesting that stage 4S neuroblastomas, which spontaneously evolve towards a differentiated phenotype, express all TGF-β receptor genes (Table 1). It is also intriguing that almost all neuroblastomas (14 out of 15 samples, 94%) with N-*myc* gene amplification (a well established marker of worst evolution) show absence of TGF-βRIII gene expression (Table 1). This result suggests a possible correlation between N-*myc* overexpression and reduced TGF-β response also in view of the observation that retinoic acid treatment of neuroblastoma cell lines causes a down-regulation of N-*myc* expression and induction of TGF-β receptor expression (Cohen et al, 1995). Future studies will be devoted to investigating the existence of this interesting relationship.

In conclusion, our studies suggest the possible occurrence of an impairment of TGF-β response in neuroblastomas of stage 3 and 4, due to specific down-regulation of TGF-βRIII gene expression. It is to be stressed that the present study confirms and remarkably extends the only previous observation showing the absence of TGF-βRIII in human tumours (Bristow et al, 1999), thus leading to the proposal of a completely novel mechanism for escaping TGF-β growth inhibitory activity in malignancies.

Due to the increasing relevance of TGF-β-dependent pathway aberrations in human tumorigenesis, further studies are required to evaluate the level and activity of other components, particularly DPC4 (SMAD4) and SMAD2 (White, 1998), in human neuroblastomas.

© 2000 Cancer Research Campaign

### ACKNOWLEDGEMENTS

This work is supported by Fondazione Italiana per la Ricerca sul Cancro (FIRC), by Associazione Italiana per la Ricerca sul Cancro (AIRC), by MURST and by Associazione Italiana per la Lotta al Neuroblastoma.

### REFERENCES

Bristow RE, Baldwin RL, Yamada SD, Kore M and Karlan BY (1999) Altered expression of transforming growth factor-beta ligands and receptors in primary and recurrent ovarian carcinoma. *Cancer* **85**: 658–668

Brodeur GM, Pritchard J, Berthold F, Carlsen NL, Castel V, Castelberry RP, De Bernardi B, Evans AE, Favrot M and Hedborg F (1993) Revisions of the international criteria for neuroblastoma diagnosis, staging and response to treatment. *J Clin Oncol* **8**: 1466–1477

Chen C, Wang X-F and Sun LZ (1997) Expression of transforming growth factor β (TGFβ) type III restores autocrine TGFβ1 activity in human breast cancer MCF-7 cells. *J Biol Chem* **272**: 12862–12867

Coffey RL, Kost LJ, Lyons RM, Moses HL and LaRusso NF (1987) Hepatic processing of transforming factor β in the rat. *J Clin Invest* **80**: 750–757

Cohen PS, Letterio JJ, Gaetano C, Chan J, Matsumoto K, Sporn MB and Thiele CJ (1995) Induction of transforming growth factor beta 1 and its receptors during all-trans-retinoic acid (RA) treatment of RA-responsive human neuroblastoma cell lines. *Cancer Res* **55**: 2380–2386

Filmus J and Kerbel RS (1993) Development of resistance mechanisms to the growth-inhibitory effects of transforming growth factor-β during tumor progression. *Curr Opin Oncol* **5**: 123–129

Filmus J, Zhao J and Buick RN (1992) Overexpression of H-ras oncogene induces resistance to the growth inhibitory action of the transforming growth factor beta-1 (TGF-β1) and alters the number of TGF-β1 receptors in rat intestinal epithelial cell clones. *Oncogene* **7**: 521–526

Fynan TM and Reiss M (1993) Resistance to inhibition of cell growth by transforming growth factor-β and its role in oncogenesis. *Crit Rev Onc* **4**: 493–540

Heldin CH, Miyazono K and Dijke P (1997) TGF-β signaling from cell membrane to nucleus through SMAD proteins. *Nature* **390**: 465–471

Hougaard S, Norgaard P, Abrahamsen N, Moses HL, Spang-Thomsen M and Skovgaard Poulsen H (1999) Inactivation of the transforming growth factor beta type II receptor in human cell lung cancer cell lines. *Br J Cancer* **79**: 10065–1011

Iolascon A, Giordani L, Moretti A, Tonini GP, Lo Cunsolo C, Mastropietro S, Borriello A and Della Ragione F (1998*a*) Structural and functional analysis of cyclin dependent kinase inhibitor genes (CDKN2A, CDKN2B and CDKN2C) in neuroblastoma. *Pediatr Res* **43**: 139–144

Iolascon A, Giordani L, Moretti A, Basso G, Borriello A and Della Ragione F (1998*b*) Analysis of CDKN2A, CDKN2B, CDKN2C and cyclin D genes status in hepatoblastoma. *Hepatology* **27**: 989–995

Iolascon A, Lo Cunsolo C, Giordani L, Cusano R, Mazzocco K, Boumgartner M, Ghisellini P, Faienza MF, Boni L, De Bernardi B, Conte M, Romeo G and Tonini GP (1998*c*) Interstizial and large 1p deletion occurs in localized and disseminated neuroblastomas and predict an unfavorable outcome. *Cancer Lett* **130**: 83–92

Johnson D, Lanahan A, Buck CR, Sehagal A, Morgan C, Mercer E, Bothwell M and Chao M (1986) Expression and structure of the human NGF receptor. *Cell* **47**: 545–554

Kadin ME, Cavaille-Coll MW, Gertz R, Massagué J, Cheifetz S and George D (1994) Loss of receptors for transforming growth factor β in human T-cell malignancies. *Proc Natl Acad Sci USA* **91**: 6002–6006

Kiefer MC, Stephans JC, Crawford K, Okino K and Barr PJ (1990) Ligand-affinity cloning and structure of a cell surface heparin sulfate proteoglycan that binds basic fibroblast growth factor. *Proc Natl Acad Sci USA* **87**: 6985–6989

Kimchi A, Wang X-F, Weinberg RA, Cheifetz S and Massague J (1988) Absence of TGF-β receptors and growth inhibitory responses in retinoblastoma cells. *Science* **240**: 196–199

Lòpez-Casillas F, Cheifetz S, Doody J, Andres JL, Lane WS and Massagué J (1991) Structure and expression of the membrane proteoglycan betaglycan, a component of the TGF-β receptor system. *Cell* **67**: 785–795

Lòpez-Casillas F, Wrana JL and Massagué J (1993) Betaglycan presents ligand to the TGFβ signaling receptor. *Cell* **73**: 1435–1444

Lu S-L, Akiyama Y, Saitoh K and Yuasia Y (1995) Mutations of the transforming growth factor-β type II receptor gene and genomic instability in hereditary nonpolyposis colorectal cancer. *Biochem Biophys Res Commun* **216**: 452–457

MacDonald RG, Pleffer SR, Coussens L, Tepper MA, Brocklebank CM, Mole JE, Anderson JK, Chen E, Czech MP and Ullrich A (1988) A single receptor binds both insulin-like growth factor II and mannose-6-phosphate. *Science* **239**: 1134–1137

Maniatis T, Fritsh E, Sambrook J (1990) Molecular cloning. In: *Cold Spring Harbor Laboratory, A Laboratory Manual*, pp. 7.6–7.22, 9.16–9.23. Cold Spring Harbor: New York

Markowitz S, Wang J, Myeroff L, Parson R, Sun L-Z, Lutterbugh J, Fan RS, Zborowska E, Vogelstein B, Brattain M and Wilson JKV (1995) Inactivation of the type II TGF-β receptor in colon cancer cells with microsatellite instability. *Science* **268**: 1336–1338

Matoba H, Sagano S, Yamaguchi N and Miyachi Y (1998) Expression of transforming growth factor-beta 1 and transforming growth factor-beta type-II receptor mRNA in papillary thyroid carcinoma. *Hormone Metab Res* **30**: 624–628

Morgan DD, Edman JC, Standring DN, Fried VA, Smith MC, Roth RA and Rutter WJ (1987) Insulin-like growth factor II receptor as a multifunctional binding protein. *Nature* **329**: 301–307

Pinkerton CR (1993) A clinical perspective in human neuroblastoma: recent advances in clinical and genetics analysis. In: *Neuroblastoma in the 1990s*, Schwab M, Tonin GP and Benard J (eds), pp. 3–10. Harwood Academic: Chur. Switzerland

Polyak K (1996) Negative regulation of cell growth by TGFβ. *Biochim Biophys Acta* **1242**: 185–199

Radeke MJ, Misko TP, Hsu C, Herzenberg LA and Shooter EM (1987) Gene transfer and molecular cloning of the rat nerve growth factor receptor. *Nature* **325**: 593–597

Royuela M, De Miguel MP, Bethencourt FR, Sanchez-Chapado M, Fraile N and Paniagua R (1998) Transforming growth factor beta 1 and its receptor types I and II. Comparison in human prostate, benign prostatic hyperplasia and prostatic carcinoma. *Growth Factors* **16**: 101–110

Saunders S, Jalkanen M, O'Farrell S and Bernfield M (1989) Molecular cloning of syndecan, an integral membrane proteoglycan. *J Cell Biol* **108**: 1547–1556

Siegel SE and Sato JK (1986) Neuroblastoma. In: *Oncology*, Moossa AR, Robson MC and Schimpfl SC (eds), pp. 1211–1231. Williams and Wilkins: Baltimore

Sun LZ, Wu G, Willson JKV, Zborowska E, Yang J, Rajkarunanayake I, Wang J, Gentry LE, Wang XF and Brattain MG (1994) Expression of transforming growth factor β type II receptor leads to reduced malignancy in human breast cancer MCF-7 cells. *J Biol Chem* **269**: 26449–26455

Wang X-F, Lin HY, Ng-Eaton E, Downward J, Ldish HF and Weinberg RA (1991) Expression cloning and characterization of the TGF-β type III receptor. *Cell* **37**: 797–805

Wang J, Han W, Zbrowska E, Liang J, Wang X, Willson JKV, Sun LZ and Brattain MG (1996) Reduced expression of transforming growth factor b type I receptor contributes to the malignancy of human colon carcinoma cells. *J Biol Chem* **271**: 17366–17371

White RL (1998) Tumor suppressor pathways. *Cell* **92**: 591–592

 *© 2000 Cancer Research Campaign*
